# Role of Point-of-Care Ultrasound (POCUS) in Cardiac Arrest: A Case Report

**DOI:** 10.7759/cureus.75996

**Published:** 2024-12-19

**Authors:** Janete Henriques, Inês Pestana, Luís Pedro, José Sousa, Humberto Machado

**Affiliations:** 1 Anesthesiology, Intensive Care and Emergency Department, Centro Hospitalar Universitário de Santo António, Porto, PRT

**Keywords:** cardiac arrest, non-defibrillable rhythms, pocus, pulmonary embolism, resuscitation

## Abstract

Acute pulmonary embolism (PE) is a significant cause of cardiac arrests, with subsequent high mortality worldwide. Early recognition of acute PE allows earlier diagnosis, stabilization, and risk stratification, which are crucial in deciding the most adequate treatment option. However, diagnosis is sometimes difficult due to nonspecific clinical presentation. We report a case of successful cardiopulmonary resuscitation with the identification of this reversible cause of cardiac arrest using point-of-care ultrasound in cardiopulmonary arrest (POCUS-CA), thus allowing appropriate prompt life-saving treatment.

## Introduction

Acute pulmonary embolism (PE) is a significant cause of mortality worldwide. It is the third most common cause of cardiovascular death among hospitalized patients in the Western world following acute myocardial infarction and stroke [1,2]. Early diagnosis and intervention have contributed to a decrease in mortality and improved outcomes [3]. Point-of-care ultrasound (POCUS) is a rapidly developing and versatile technology that can improve patient care by providing real-time clinical information. With appropriate training, it can be an invaluable tool in critical care medicine and extremely helpful in emergent scenarios, namely cardiopulmonary resuscitation (CPR) [4]. It allows for the identification of cardiac activity and potentially reversible causes of cardiac arrest, in addition to monitoring the efficacy of chest compressions, thus improving the quality of CPR. This case report illustrates the relevance and advantages of using POCUS in CPR.

This article was previously presented as a meeting abstract at the 2023 O Norte da Anestesia on November 17, 2023.

## Case presentation

A 63-year-old man, with a history of obesity, dyslipidemia, arterial hypertension, and acquired familial amyloidosis polyneuropathy (FAP) with both cardiac and dysautonomic involvement, underwent orthotopic liver retransplantation due to FAP.

On the first postoperative day, due to hepatic artery thrombosis, the patient underwent thrombectomy and arterial anastomosis reconstruction, and treatment with unfractionated heparin was initiated. Two days later, he developed hemorrhagic shock and required emergency surgery as well as the administration of 10 units of packed red blood cells. Consequently, anticoagulation was discontinued. He spent 13 days in the intensive care unit (ICU) and was later transferred to the ward.

**Figure 1:**
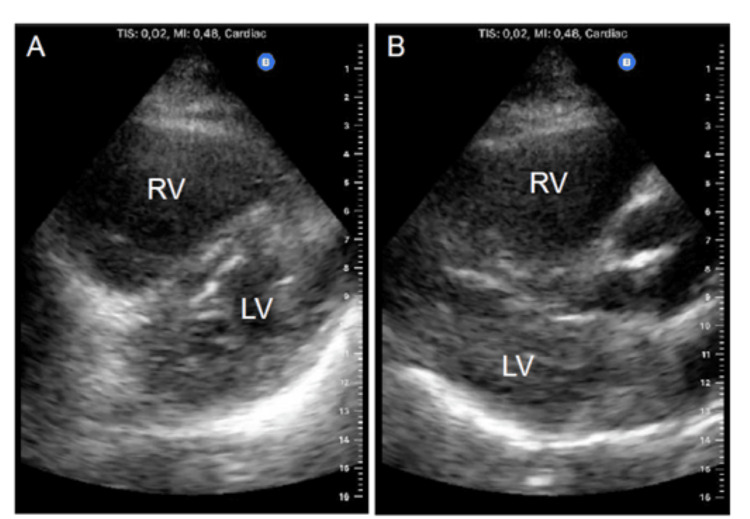
POCUS in cardiac arrest. (A) Parasternal short axis; (B) parasternal long axis. Images were obtained using a Butterfly iQ™ probe. POCUS: point-of-care ultrasound; RV: right ventricle; LV: left ventricle.

The patient suffered a sudden collapse on the first ward day. Immediate CPR was initiated by the attending team, and the Intrahospital Emergency Team (IET) was activated. Upon IET arrival, pulseless electrical activity was detected, and 1 mg of adrenaline was administered. Return of spontaneous circulation (ROSC) was achieved at the second rhythm analysis, and the airway was secured with an endotracheal tube. During CPR, right before rhythm analysis, point-of-care cardiac ultrasound using a portable ultrasound machine (Butterfly™) was performed by the most experienced operator using a parasternal view (Figure 1A, B).

It revealed dilation of the right heart chambers, raising the suspicion of a PE as the etiology for the CA, which was further supported by the reduced end-tidal CO2. The patient was transported to the emergency room (ER) for stabilization and post-cardiac arrest care, and the Cardiology team was immediately contacted for a formal transthoracic echocardiogram (TTE) given the suspicion of PE. The TTE revealed severe right ventricular dilation with severe systolic dysfunction.

**Figure 2:**
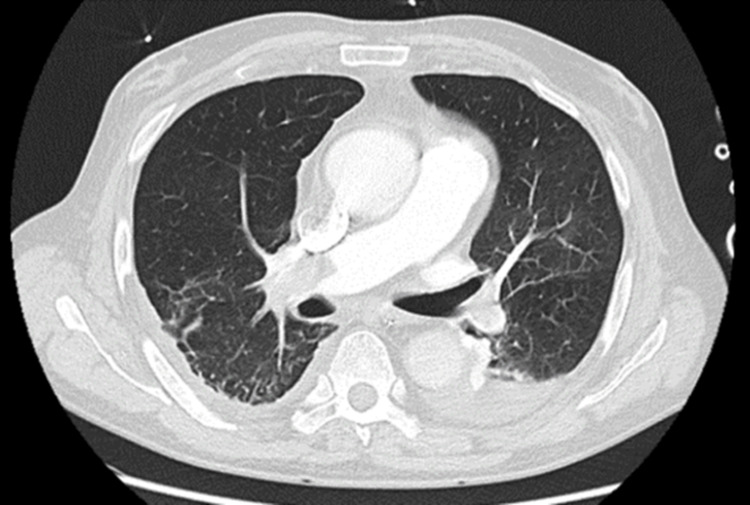
Massive PE on angio-CT. PE: pulmonary embolism.

Subsequently, a chest CT angiography was performed (Figure 2), revealing a bilateral massive thrombus of the pulmonary artery with almost complete obstruction of the right lumen, confirming the suspicion of a massive central PE. Thrombolysis was contraindicated in this context, so after multidisciplinary discussion, an emergent pulmonary thrombectomy was performed, achieving successful reperfusion of both lungs. The patient was readmitted to the ICU, and after an 11-day ICU stay, he was transferred to the ward and was discharged home 24 days later. 

## Discussion

Cardiac arrest requires rapid, targeted interventions and resuscitative efforts. When approaching a patient in cardiac arrest with non-defibrillable rhythms (pulseless electrical activity and asystole), it is a priority to consider the diagnosis of potentially reversible causes. These causes are organized into the mnemonic of “Hs and Ts,” presented in Table 1.

**Table 1:** Reversible causes of cardiac arrest.

4Hs	4Ts
Hypoxia	Tension pneumothorax
Hypokalemia or hyperkalemia and other electrolyte disorders	Tamponade
Hypothermia	Thrombosis (coronary and pulmonary)
Hypovolemia	Toxins

With increasing portability and accessibility, POCUS has become a safe, accurate, and invaluable diagnostic tool for critically ill patients in various settings, namely in emergent scenarios [5].

Although resuscitation algorithms do not mandate the use of POCUS, it is currently recognized in resuscitation guidelines and has become standard practice in many emergency departments. POCUS-CA is a highly versatile tool, as it can be used to identify cardiac activity, efficacy of cardiac compressions, and diagnose potentially reversible causes, the latter allowing early decision-making and possibly affecting outcome and overall survival [3,5]. POCUS has the greatest utility in non-shockable rhythms by identifying reversible causes, which represent the majority of in-hospital cardiopulmonary arrests [6]. Etiologies such as tension pneumothorax, cardiac tamponade, hypovolemia, and myocardial ischemia are all well suited for a diagnostic evaluation with ultrasound.

Manual pulse checks during cardiac arrest lack accuracy and can prolong pauses, as suggested by evidence [1]. Ultrasound can also be useful in differentiating true pulseless electrical activity (PEA) from pseudo-pulseless electrical activity. PEA consists of an organized rhythm with no palpable pulse or detectable contractility on cardiac ultrasound. Pseudo-PEA is distinguished by a preserved, organized cardiac contractility, despite its inability to generate a detectable pulse. The latter is associated with an increased likelihood of achieving return of spontaneous circulation (ROSC) during ACLS [6].

In our case, POCUS was used both during rhythm analysis to guide CPR and after ROSC. Identification of echographic findings suggestive of PE, namely right ventricular and atrial enlargement, heightened our suspicion of acute PE and facilitated prompt communication with the Cardiology Department for a formal evaluation and appropriate treatment. When performing cardiac ultrasound, it is important to differentiate acute strain from chronic right heart pressure, which is generally seen in conditions of chronic pulmonary arterial hypertension. In the latter case, the right ventricular wall will compensate by hypertrophy. In contrast, in acute right ventricular tension, the chamber typically has a thin wall measuring less than 5 mm [5].

Irrespective of the cause, timely and adequate CPR is vital, with high-quality chest compressions being the most critical component of CPR. Additionally, it is of utmost importance to reduce the duration of pauses between compression cycles, which should not exceed 10 seconds. The balance between obtaining diagnostic information via POCUS and ensuring uninterrupted CPR is critical for optimal patient outcomes [3,6]. Although several guidelines have suggested a role for POCUS in cardiac arrest, there are concerns that POCUS may lead to prolonged interruptions in chest compressions.

Gottlieb and Alerhand described several strategies to minimize prolonged interruptions, namely that the scan should be performed by the most experienced sonographer, who should not be burdened with the responsibilities of team leading and also that the transducer should be placed on the chest to identify the optimal cardiac window prior to pausing compressions [1].

In our case, the presence of enough team members during resuscitation enabled cardiac POCUS to be performed by the most experienced sonographer who was not involved in team leading. Also, the transducer was placed prior to pausing compressions to minimize interruptions. Furthermore, the images were acquired during pulse check and analyzed after resuming CPR, in line with the literature concerning POCUS-CA [5].

Although it is recommended to start POCUS-CA evaluation with the subxiphoid window to obtain a four-chamber view, we performed a parasternal view due to surgical dressings. This is also a valid option, though less employed, and has been included in several cardiac arrest ultrasound protocols [5].

Focusing on treatment options for massive PE, guidelines suggest systemic thrombolysis in intermediate-risk PE and severe right ventricle dysfunction/clinical deterioration when bleeding risk is low. On the other hand, patients with intermediate to high-risk PE and contraindications for thrombolysis or high bleeding risk should be considered for catheter thrombectomy, which was the case of our patient, who was submitted to a recent major abdominal surgery and a postoperative hemorrhagic shock [2].

## Conclusions

POCUS is a valuable tool for identifying reversible causes of cardiac arrest and guiding appropriate life-saving treatment. In experienced hands, POCUS could be integrated into ACLS algorithms to provide prompt and targeted management for critically ill patients. Implementing POCUS safely without delaying CPR is possible by taking the necessary precautions and ensuring that the sonographer has adequate expertise, as demonstrated in this case report.
